# Feasibility and SNR Performance of Hyperpolarized ^129^Xe Gas Exchange Imaging Using a Balanced SSFP Sequence

**DOI:** 10.1002/mrm.70503

**Published:** 2026-07-05

**Authors:** Agilo L. Kern, Marcel Gutberlet, Jens M. Hohlfeld, Frank Wacker, Jens Vogel‐Claussen

**Affiliations:** ^1^ Institute for Diagnostic and Interventional Radiology, Hannover Medical School Hannover Germany; ^2^ Biomedical Research in Endstage and Obstructive Lung Disease Hannover (BREATH) German Center for Lung Research (DZL) Hannover Germany; ^3^ Department of Radiology Charité – University Medical Center Berlin, Corporate Member of Freie Universität Berlin and Humboldt‐Universität zu Berlin Berlin Germany; ^4^ Clinical Airway Research Fraunhofer Institute for Toxicology and Experimental Medicine (ITEM) Hannover Germany; ^5^ Department of Respiratory Medicine and Infectious Diseases Hannover Medical School Hannover Germany

**Keywords:** ^129^Xe, bSSFP, dissolved phase, gas exchange, hyperpolarization, lung

## Abstract

**Purpose:**

To develop a balanced SSFP (bSSFP) sequence for pulmonary ^129^Xe dissolved‐phase imaging based on a previously described double‐echo sequence facilitating separation into red blood cell and membrane signals, to test its feasibility and compare its SNR performance and imaging results to an analogous FLASH sequence.

**Methods:**

12 healthy volunteers underwent hyperpolarized ^129^Xe MRI with both sequences with two different choices of flip angle for FLASH. SNR was quantified using a pseudo multiple replica method, normalized by ^129^Xe polarization and ventilated volume. Chemical shift separation was performed in k‐space taking results from time‐domain fits of signal evolution as input, either approximating *T*_E_ as constant or taking the finite readout duration into account. Results were compared between sequences and methods by linear mixed models, Bland–Altman, and scatter plots.

**Results:**

Normalized SNR of the first echo was significantly greater for bSSFP compared to FLASH with the same flip angle, *p* = 0.003, but not with lower flip angle resulting in equivalent depolarization, *p* = 0.98. The effective number of signal averages in signal decomposition was significantly higher compared to FLASH with both flip angles and both dissolved resonances, *p* < 0.008. Neglecting the finite readout duration was found to introduce bias of derived RBC‐M ratios but prevent noise amplification.

**Conclusion:**

Hyperpolarized ^129^Xe dissolved‐phase imaging using dual‐echo bSSFP is feasible and provides superior effective number of signal averages resulting in improved SNR efficiency of signal decomposition. Future work should concentrate on the application in patients with lung disease.

## 1 Introduction

Hyperpolarized ^129^Xe MRI has emerged as a versatile tool for the assessment of lung function in patients with lung disease. Imaging the distribution of inhaled ^129^Xe allows for the detection of local ventilation impairment in obstructive lung diseases [1]. Changes in alveolar microstructure, for example due to pulmonary emphysema, may be detected by altered apparent diffusion coefficients [2].

In addition, thanks to xenon's good solubility and the wide chemical shift range, hyperpolarized ^129^Xe MRI allows for the assessment of gas uptake [3]. With suitable dynamic measurements, these data may also be used for the determination of alveolar septal structure [4]. Yet, imaging of the ^129^Xe dissolved phase is technically challenging due to the low amount of ^129^Xe dissolved in the lung's red blood cells (RBC) and membrane tissue (M) at a given instant of time. This results in low SNR and, consequently, low achievable image resolution, which may limit the diagnostic value.

Balanced SSFP (bSSFP), also known as TrueFISP, balanced FFE or FIESTA, is thought to be the sequence with highest SNR per unit time among all known sequences [5]. bSSFP has been found to provide significant gains compared to FLASH sequences in imaging of hyperpolarized gases [6]. Yet, due to short transverse relaxation times of the ^129^Xe dissolved phase in well vascularized tissues [7], potential SNR gains in the dissolved phase can be expected to be smaller. Additionally, blood flow and exchange between dissolved and gaseous ^129^Xe as well as between ^129^Xe in RBC and M complicate the evolution of magnetization, making numerical predictions difficult. Nonetheless, even a small SNR gain may be relevant given the typically low SNR of dissolved‐phase images.

A well‐known limitation of bSSFP is its sensitivity to off‐resonance which may be partly mitigated by short repetition times. Although most implementations for ^129^Xe gas‐exchange imaging based on FLASH sequences use waiting times for replenishment of ^129^Xe signal [8], previous work has suggested that equivalent results for dissolved‐phase ratios may be obtained at shorter *T*_R_ by appropriately reducing the flip angle [9, 10], which also has the advantage of reducing longitudinal relaxation when keeping the number of excitations fixed.

The ^129^Xe dissolved phase consists of at least two resonances in humans in the lung and multiple methods have been used in the literature for imaging them separately. The one‐point Dixon method for separation uses a single readout after excitation with an echo time chosen such that RBC and M are out of phase by approximately 90°, as estimated by a calibration scan in the whole lung. This method is well established [8] but may also suffer from biases of various kinds [11]. Alternatively, multi‐echo acquisitions combined with IDEAL employing a least‐squares fit or similar techniques have been used for robust separation of the signals [12, 13, 14]. This poses restrictions on the minimum *T*_R_ achievable using the sequence. In the field of hyperpolarized ^13^C imaging, Leupold et al. previously demonstrated the utility of multi‐echo bSSFP with tailored repetition time to avoid banding at any of the resonances [15].

A previous preliminary study has investigated the utility of a single‐echo bSSFP sequence for ^129^Xe dissolved‐phase imaging in kidney and lung [16]. While an SNR improvement was reported in the kidneys compared to a FLASH sequence, reduced SNR was reported in the lungs. This was not based on a statistical comparison, however, and there may be differences between acquisitions that are difficult to control experimentally, like, for example, lung inflation level.

A sequence allowing for minimal *T*_R_ while collecting at least two echoes for robust chemical shift separation of RBC and M is based on a half‐radial double echo. In the field of proton imaging of the lung, a half‐radial double echo bSSFP sequence termed bSTAR has been described for structural imaging [17].

Purpose of this study was to develop a bSSFP sequence for ^129^Xe gas‐exchange imaging based on this previously described double‐echo sequence, to test its feasibility and to rigorously compare its SNR performance as well as imaging results to an analogous FLASH sequence with identical trajectory and timing.

## 2 Methods

### 2.1 Dual‐Echo bSSFP Sequence

**FIGURE 1:**
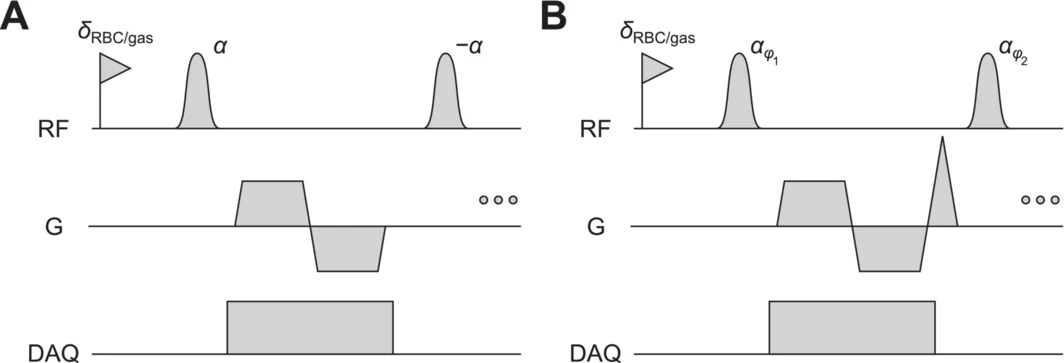
Sequence diagram of (A) dual‐echo bSSFP sequence. The system frequency is adjusted to either the chemical shift of ^129^Xe in RBCs or to the chemical shift of gaseous ^129^Xe at 0 ppm. Repetition of the entire sequence at both frequencies facilitates normalization of dissolved ^129^Xe signals to the gas phase. Excitation phase is alternated between repetitions and gradient moment is balanced. (B) In the FLASH version of the sequence, excitation phase is rotated and a spoiling gradient with maximal gradient moment added, respecting limits on slew rate and sequence timing. Two different dissolved‐phase flip angles were used as detailed in the main text. The flip angle was reduced for gas‐phase acquisition in the FLASH sequence to avoid too strong depolarization. Abbreviations: bSSFP, balanced SSFP; DAQ, data acquisition; G, gradient; RBC, red blood cell.

The sequence was implemented according to the sequence diagram shown in Figure 1. No dephasing is applied after excitation, corresponding to a first “echo” in the sense of a start in the k‐space center. The first half of the bipolar readout gradient corresponds to a half‐radial trajectory outward, the second half to a reversed half‐radial trajectory inward and formation of a second echo. The *T*_R_ was chosen as 2.91 ms, which is the inverse of the frequency separation of ˜343 Hz between RBC and M at 1.5 T. Excitation was performed at 3826 Hz downfield from the gas resonance corresponding to the RBC frequency such that RBC and M are in neighboring bSSFP pass bands. The duration of a previously described [4] RF pulse was adjusted to 1024 μs, which was found to minimize excitation of gaseous ^129^Xe in preliminary phantom experiments. The echo times chosen were 0.62 and 2.08 ms resulting in a ˜180° phase change of ^129^Xe in M. Initial 150 acquisitions without spatial encoding were used. The first 100 acquisitions were discarded to ensure steady‐state magnetization. The remaining 50 acquisitions were used to determine the time evolution of magnetization and to obtain estimates of chemical shifts and transverse relaxation rates. The end points of the radial trajectory followed a three‐dimensional tiny golden angle trajectory using the generalized golden means ϕ_13,1_ and ϕ_13,2_ [18] mapped to a full sphere. The trajectory was scaled to achieve 1 cm voxel size.

3217 radial spokes were acquired corresponding to Nyquist sampling of a 32 × 32 × 32 matrix. Both echoes were acquired using a single highly oversampled data acquisition period of 600 points at a dwell time of 2.5 μs. Data acquisition started before the beginning and ended after the end of gradient activity. The first three and the last 15 points were discarded during reconstruction. At the end of the image acquisition, a single free‐induction decay was acquired at a dwell time of 25 μs using the same excitation RF pulse as for imaging.

The flip angle was chosen to be 13.3°. This choice was based on simulations suggesting maximum SNR for the used reconstruction method in acquisitions with remaining magnetization roughly 30% of the initial value and preliminary observations of the signal decay in healthy volunteers. The entire sequence was repeated at the gas frequency with otherwise identical parameters to facilitate local normalization of signals to the gas phase.

In addition, a FLASH version of the sequence was created by adding phase cycling as well as a spoiler gradient to the sequence, Figure 1. This sequence was tested with two different flip angles acting on the dissolved phase as detailed below. The flip angle for gas‐phase acquisition was set to 1.6° for FLASH in both cases.

### 2.2 Volunteer Study

The institutional review board of Hannover Medical School approved the study for sequence comparison in healthy human volunteers (#11943‐BO‐S‐2025) and all subjects gave written informed consent. Preliminary investigations for sequence development and optimization of sequence parameters were made in a separate study (#7685). Exclusion criteria were known lung disease, lung surgery, recent lung infection as well as general MRI contraindications.

The study consisted of two study parts comparing (A) bSSFP and FLASH sequences with the same high dissolved‐phase flip angle 13.3° and (B) bSSFP with high flip angle and FLASH with low flip angle 9° chosen such that the magnetization decay was approximately the same for both sequences. The second choice should ensure that image sharpness and noise amplification during reconstruction are as comparable as possible between bSSFP and FLASH. Subjects were alternatingly allocated to one of both study parts and underwent hyperpolarized ^129^Xe MRI as well as lung function testing (SpiroScout, Ganshorn Medizin Electronic GmbH, Niederlauer, Germany).

Imaging was performed at 1.5 T (Avanto, Siemens Healthcare GmbH, Erlangen, Germany) using a custom‐made circularly polarized birdcage coil tuned to 17.6 MHz for both transmit and receive (Rapid Biomedical GmbH, Rimpar, Germany). After subject positioning in the supine position and acquisition of scout images, noise measurements were performed by running the sequences prior to inhalation of ^129^Xe.

Hyperpolarized xenon was authorized as a medical device of in‐house production. Three batches of hyperpolarized xenon were produced (Model 9810, Polarean Inc., Durham, NC) using a gas mixture containing isotopically enriched ^129^Xe (87%, Linde GmbH, Pullach, Germany) for each scan session. The first batch was used for transmitter calibration using the magnitude‐decay method [19]. The second and third batches were used for gas‐exchange imaging using dual‐echo bSSFP and FLASH, respectively, with the order of sequences alternating between subjects. In both cases, 600 mL of ^129^Xe was collected, dispensed in Tedlar bags (Restek GmbH, Bad Homburg vor der Höhe, Germany), and topped up using nitrogen to achieve a total volume of 1 L. Polarization levels were measured (Model 2881, Polarean Inc., Durham, NC) after dispensing and immediately before application for SNR normalization. Subjects inhaled the doses starting from functional residual capacity. Dissolved and gas acquisitions were made in a single 20 s breathhold.

### 2.3 SNR Quantification

Image reconstruction and analysis was performed in GNU Octave. SNR was quantified for each data set using the pseudo multiple replica method of Robson et al. [20]. Synthetic noise intensity was determined from the previously described noise measurements after excluding outliers occurring predominantly during gradient switching and was additionally normalized to the value at Larmor frequency as described by Kellman et al. [21]. 10^4^ replicas of the image volume corresponding to the first echo of both sequences were reconstructed and SNR maps obtained by division by the standard deviation of the real part of the image stack.

Density compensation factors were determined assuming the analytical relationship [22]

(1) $$D\left(k\right)\propto \frac{1}{k^{2}G\left(k\right)}$$

with *D* the sampling density and *G* the gradient amplitude at k‐space location *k*. Data were additionally apodized using a von Hann window. This is necessary to prevent excessive noise in outer regions of k‐space arising from chemical shift separation as described below. For SNR comparison, images were reconstructed using the adjoint non‐uniform fast Fourier transform (NUFFT) algorithm of the Berkeley Advanced Reconstruction Toolbox (version 0.9.00 [23]) as a linear reconstruction method facilitating use of the pseudo multiple replica method [20].

An exponential decay as a function of projection number was fitted to the magnitude of the raw data in the center of k‐space for data of the imaging part of the sequence. The decay function was normalized to its first point and its inverse used as a temporal basis in the NUFFT algorithm to prevent blurring associated with polarization decay. This should ensure that the sequence variants are reconstructed in a way resulting in point‐spread functions as similar as possible to facilitate a fair comparison.

A lung mask was semi‐automatically generated by thresholding of the SNR maps and subsequent manual refinement by AK (10 years of experience in lung MRI). SNR was then quantified as the median of the real part of phase‐corrected SNR values within the lung mask. This averaged SNR value was normalized to a polarization level of 20% as well as to a masked volume of 5000 voxels, assuming SNR is proportional to polarization and inversely proportional to the number of voxels within the lung mask, which neglects potential effects of changes in lung microstructure.

### 2.4 Dissolved‐Phase Signal Decomposition

RBC and M phases were separated using least‐squares chemical shift separation [24, 25]. In order to determine global off‐resonance frequencies *f* and apparent transverse relaxation times *T*_2_*, 50 acquisitions of bSSFP after reaching steady state and without spatial encoding were averaged into 5 sets of 10 acquisitions. A model function describing a sum of two complex exponentials corresponding to RBC and M signals as function of echo time was fitted to the data. The median of the results for off‐resonance frequencies *f* and apparent transverse relaxation rates *T*_2_* was used to create chemical‐shift encoding matrices.

(2) $$E_{m,n,q}=e^{\left(2\pi if_{q}-{1/T}_{2}^{*}\right)T_{E,m,n}}$$

similar to [25], where *q* denotes either RBC or M and *m* the first or second half of the readout, respectively.

Two reconstructions were performed: one accounting for the change of echo time for different pairs of readout points *n* due to finite readout duration (exact solution) and a second one approximating *T*_E_ as constant and assuming the value at *k* = 0 during the whole readout. The Moore–Penrose inverse of the chemical shift encoding matrix was calculated and multiplied with the raw data to separate signals in k‐space as previously described [25]. For this purpose, the second half of the readout was reflected. For the exact solution, matrix inversion was performed for each pair *n* of readout points at the same position in k‐space individually. The k‐space trajectory is symmetric by design, but there may exist minor shifts between pairs of readout points due to finite dwell‐times and potential gradient delays, which was addressed by high oversampling as described before and careful tuning of the delay in order to minimize differences in k‐space support or weighting between echoes.

Finally, the effective number of signal averages NSA for both RBC and M was calculated as follows:

(3) $${\mathrm{NSA}}_{q}=\frac{1}{{\left(\mathrm{inv}\left(E^{†}E\right)\right)}_{q,q}}$$

with † the Hermitian conjugate. This quantity is reflective of the SNR efficiency of the encoding in the center of k‐space for the exact solution and the whole images for the constant *T*_E_ approximation.

In the acquisition at the gas frequency, both echoes were averaged to facilitate calculation of ratio maps. In the case of the FLASH acquisition, gas‐phase data were multiplied by a correction factor of 5.6 given by the ratio of sine functions of flip angles to take the reduced flip angle in the gas phase into account. Whole lung averages of dissolved‐phase ratios were calculated by summing the respective signals within the previously described mask covering the whole lung and subsequently forming the ratio of sums.

### 2.5 Magnetization Decay Correction

For correction of the decay of magnetization during dissolved‐phase acquisition leading to inhomogeneous polarization in the subsequent gas‐phase acquisition during the same breathhold by xenon polarization transfer contrast [26], linear subspace reconstruction was performed [27] which was previously used for mapping of relaxation times in conventional proton imaging and is applied to ^129^Xe MRI here. This reconstruction procedure models signal decay similarly as in [28] and allows for spatially varying decay rates by projection onto a set of linearly independent basis vectors. A dictionary in the form of a matrix with 500 mono‐exponential decays with decay constants ranging from half to twice the decay observed in the center of k‐space was created. A singular value decomposition was calculated, and the first two singular vectors were used as temporal basis for linear subspace reconstruction. Reconstruction was performed using inverse NUFFT of the Berkeley Advanced Reconstruction Toolbox, and reconstructed subspace coefficients were multiplied by the basis vectors to obtain the signal decay in each voxel. A linear function was fitted to the logarithm of the signal magnitude in each voxel to obtain a regional estimate of the magnetization decay. The coefficient of variation of local signal decay during the acquisition was calculated for comparison between sequences.

### 2.6 Statistical Analysis

Statistical analysis was performed using R (version 4.3.3). Data from both study parts were jointly analyzed using a linear mixed model (R package lmerTest, version 3.1.3) including measurement (bSSFP, FLASH with high flip angle, FLASH with low flip angle) or method (dissolved‐phase ratio from time‐domain fit, image space using constant *T*_E_ approximation or image space using exact solution) as fixed effect and subject as random effect. Pairwise contrasts between bSSFP and both FLASH variants were calculated using estimated marginal means (R package emmeans, version 1.10.0) and assessed for statistical significance after applying Dunnett‐type adjustment for multiple comparisons. Bland–Altman analysis was performed and descriptive scatter plots were created to assess agreement of dissolved‐phase ratios determined with different methods. The significance level was set at 0.05 two‐sided.

## 3 Results

A total of 12 subjects (6 male/6 female, age 27.0 ± 4.5 years) were recruited and completed all study procedures. FLASH images of the gas phase could not be evaluated in one subject of study part A since inadvertently a wrong flip angle was used in the sequence protocol. Table 1 summarizes subject demographics as well as results from lung function testing.

**TABLE 1:** Subject demographics and results from lung‐function testing.

	Age/years	Sex	FEV_1_/% of predicted value	FVC/% of predicted value	FEV_1_/FVC/%
Study part A (*n* = 6)	28.9 ± 5.2	3 M/3 F	96.7 ± 11.6	103.7 ± 5.5	78.0 ± 10.7
Study part B (*n* = 6)	25.0 ± 2.8	3 M/3 F	101.9 ± 3.0	105.7 ± 9.4	82.0 ± 6.0
All (*n* = 12)	27.0 ± 4.5	6 M/6 F	99.3 ± 8.5	104.7 ± 7.4	80.0 ± 8.5

Abbreviations: F, Female; FEV_1_, Forced expiratory volume in 1 second; FVC, Forced vital capacity; M, Male.

**FIGURE 2:**
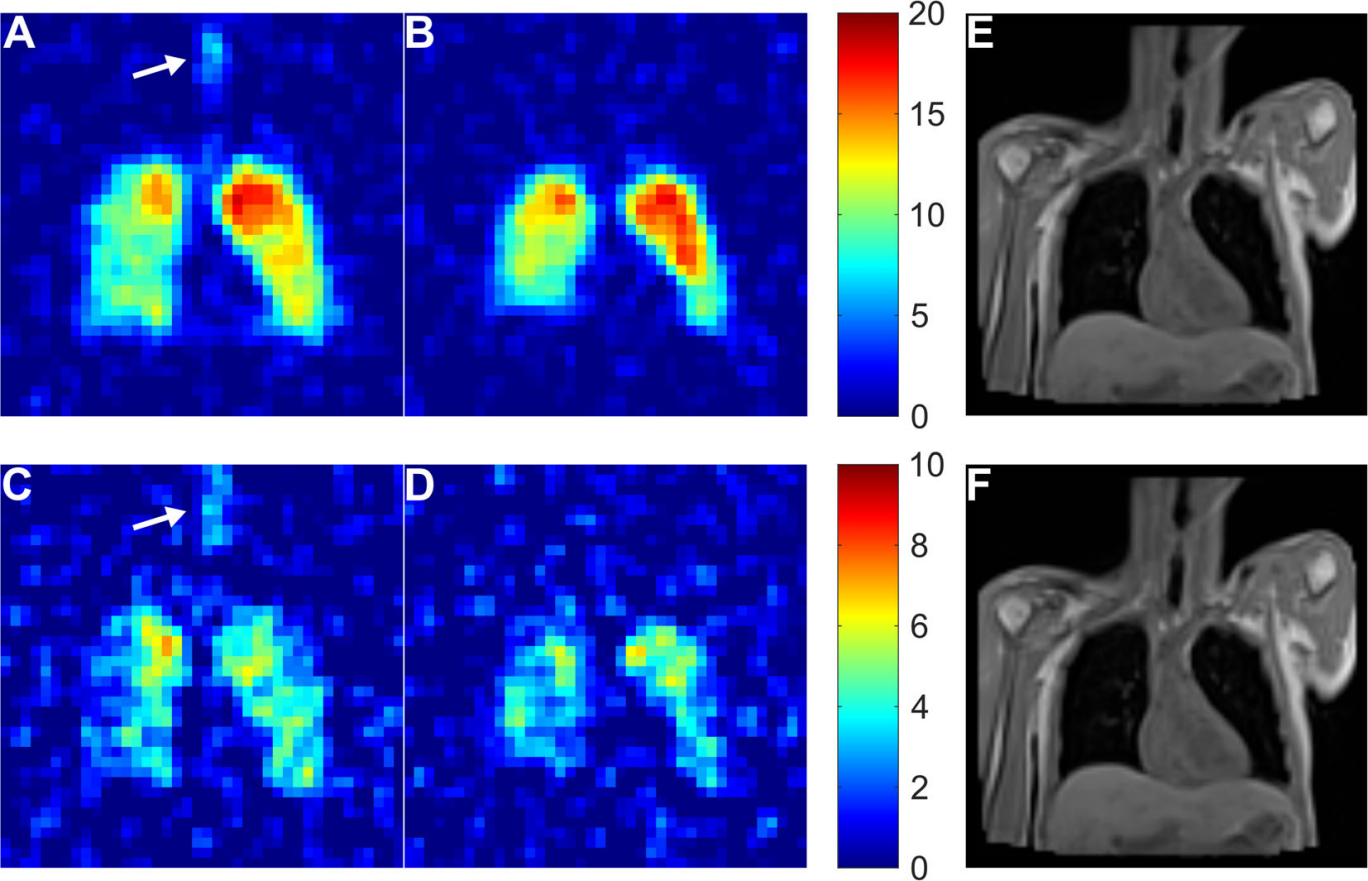
Real parts of phase‐corrected SNR maps in coronal orientation before normalization for polarization level and masked volume in a 24‐year‐old female volunteer in study part B. (A) First dissolved‐phase echo of bSSFP and (B) first dissolved‐phase echo of FLASH sequence with reduced flip angle. (C, D) show corresponding maps for the second echo. Interestingly, dissolved‐phase images show signal in the area of the larynx in bSSFP (arrows) which is absent in the FLASH image. (E, F) show a corresponding image from anatomical proton imaging after inhalation of an equivalent amount of air in similar slice location. Abbreviation: bSSFP, balanced SSFP.

Example SNR maps of both dissolved‐phase echoes and both sequences in one subject as well as an anatomical proton image for reference are shown in Figure 2. Interestingly, dissolved‐phase signal from outside the lung, specifically the larynx, becomes apparent in bSSFP which has so far not been observed in FLASH acquisitions. A comparison with corresponding gas signals reconstructed with artificial off‐resonance shows different appearance which suggests that this signal is not due to signal contamination from spurious excitation, Figure S1.

**FIGURE 3:**
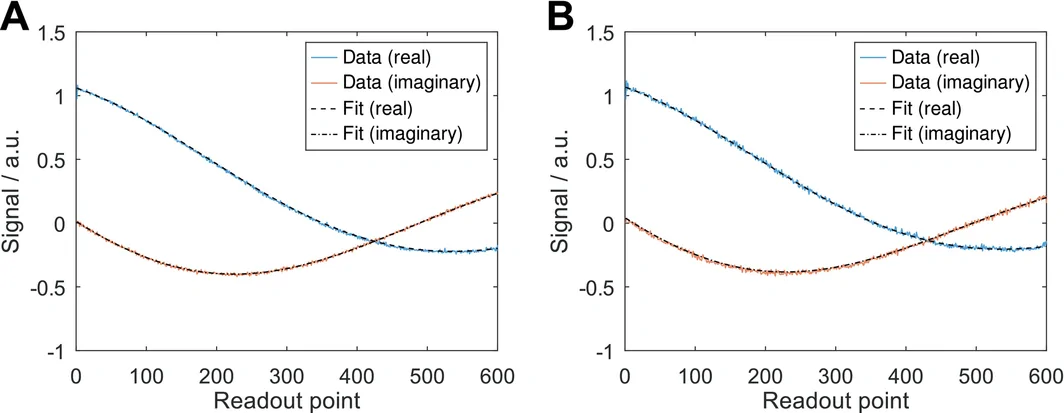
(A) Time evolution of magnetization during the bSSFP readout as well as fitted model function in a 35‐year‐old male volunteer in study part A. Data shown are an average of 50 T_R_ periods. The data is seen to be well described by the model function consisting of a sum of complex exponentials. Fitting results are $T_{2,M}^{*}=2.61\;\mathrm{ms}$, $f_{M}=3481.0\;\mathrm{Hz}$, $T_{2,\mathrm{RBC}}^{*}=2.11\;\mathrm{ms}$, $f_{\mathrm{RBC}}=3856.6\;\mathrm{Hz}$. (B) Similar results are obtained in the case of FLASH with high flip angle although with somewhat shorter transverse relaxation times, $T_{2,M}^{*}=2.35\;\mathrm{ms}$, $f_{M}=3477.5\;\mathrm{Hz}$, $T_{2,\mathrm{RBC}}^{*}=1.69\;\mathrm{ms}$, $f_{\mathrm{RBC}}=3851.7\;\mathrm{Hz}$. Abbreviations: a.u., arbitrary units; bSSFP, balanced SSFP; M, membrane; RBC, red blood cell.

Figure 3 shows the bSSFP and FLASH signal observed in the time domain during the period without spatial encoding as well as a fitted model function. The good agreement between model function and data suggest that the time evolution of magnetization is well described by a sum of exponentially decaying complex signals in both cases. Group means and SDs of the fitting results for bSSFP are $T_{2,M}^{*}=2.82\pm 0.30\;\mathrm{ms}$, $f_{M}=3478.6\pm 4.1\;\mathrm{Hz}$, $T_{2,\mathrm{RBC}}^{*}=1.99\pm 0.18\;\mathrm{ms}$, $f_{\mathrm{RBC}}=3846.4\pm 7.8\;\mathrm{Hz}$.

**FIGURE 4:**
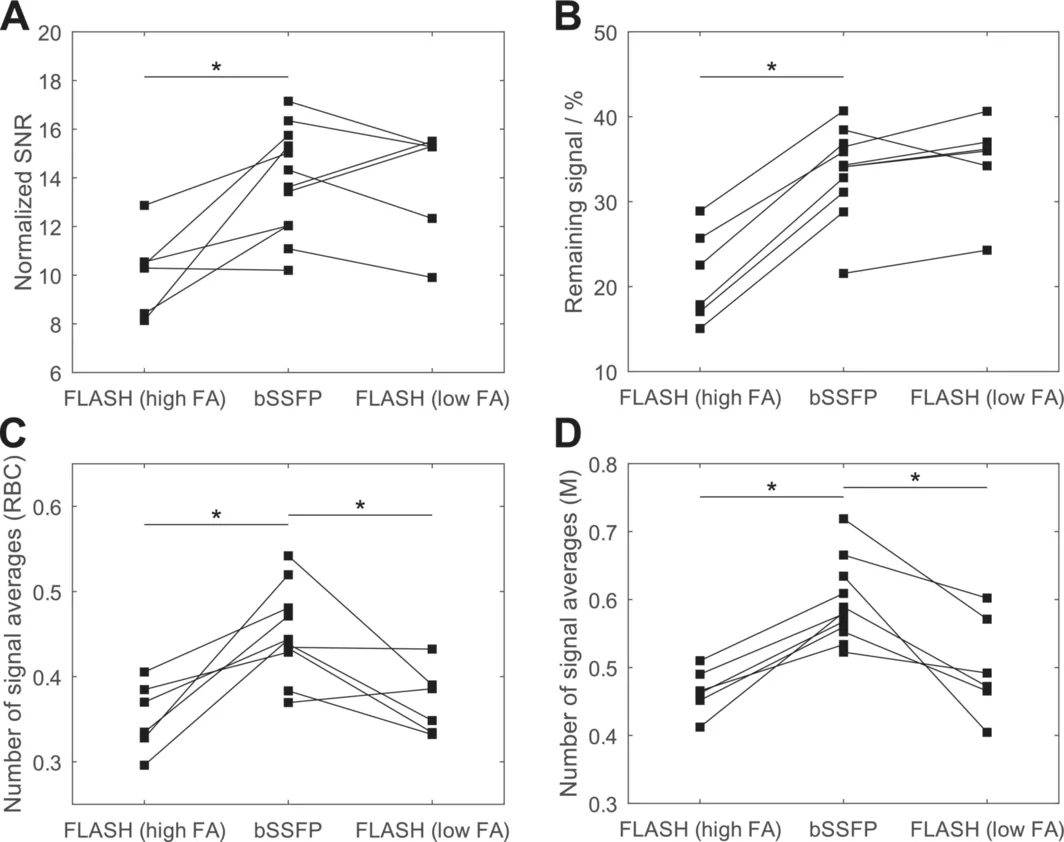
Comparison of (A) Normalized SNR in FLASH with high flip angle (FA), bSSFP and FLASH with low FA. A significant difference is observed between FLASH with high FA and bSSFP with the same FA but not between bSSFP and FLASH with low FA. (B) Remaining signal after the last excitation normalized to the signal after the first excitation of the imaging part of the sequence. Again, a significant difference is observed between FLASH with high FA and bSSFP but not for FLASH with low FA and bSSFP. (C) Effective number of signal averages of the RBC resonance after signal decomposition using the constant *T*_E_ approximation. The number is increased in bSSFP compared to both FLASH variants. (D) The same holds true in the case of the M resonance.

Normalized whole‐lung averages of SNR in the first echo, remaining magnetization at the end of the acquisition and effective number of signal averages for both RBC and M are shown in Figure 4. A significant difference of normalized SNR is observed between bSSFP and FLASH with the same flip angle, *p* = 0.003, but not between bSSFP and FLASH with reduced flip angle, *p* = 0.98. With the reduced flip angle chosen for FLASH, no significant difference is observed in the remaining signal at the end of the acquisition compared to bSSFP, *p* = 0.28, whereas choice of the same high flip angle leaded to significantly lower remaining signal, *p* < 0.001. The effective number of signal averages associated with chemical shift separation, which is primarily affected by apparent transverse relaxation times, was significantly higher for RBC in bSSFP compared to FLASH with high flip angle, *p* = 0.001, and compared to FLASH with low flip angle, *p* = 0.008. Similarly, the effective number of signal averages was significantly higher for M in bSSFP compared to FLASH with high flip angle, *p* < 0.001, and compared to FLASH with low flip angle, *p* < 0.001.

**FIGURE 5:**
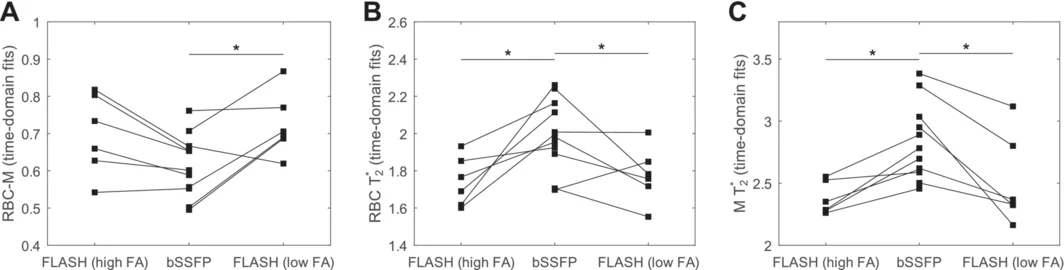
Comparison of results from the sequence variants in time‐domain fits for (A) RBC‐M. A significant difference is observed between bSSFP and FLASH with reduced flip angle while there is only a nonsignificant trend in the comparison of bSSFP and FLASH with the same high flip angle. (B, C) Apparent transverse relaxation times of ^129^Xe in RBC and M, respectively, are significantly increased in bSSFP compared to both FLASH variants.

Figure 5 shows a comparison of results from fitting model functions to signal evolution in the time domain between sequences. There was a nonsignificant trend for a reduced RBC‐M ratio in bSSFP compared to FLASH with high flip angle, *p* = 0.06, whereas there was a significant reduction compared to FLASH with low flip angle, *p* = 0.01. The apparent transverse relaxation time in RBC was significantly longer in bSSFP compared to FLASH with high flip angle, *p* = 0.01, and compared to FLASH with low flip angle, *p* = 0.04. Similarly, the apparent transverse relaxation time in M was significantly longer in bSSFP compared to FLASH with high flip angle, *p* = 0.006, and compared to FLASH with low flip angle, *p* = 0.002.

**FIGURE 6:**
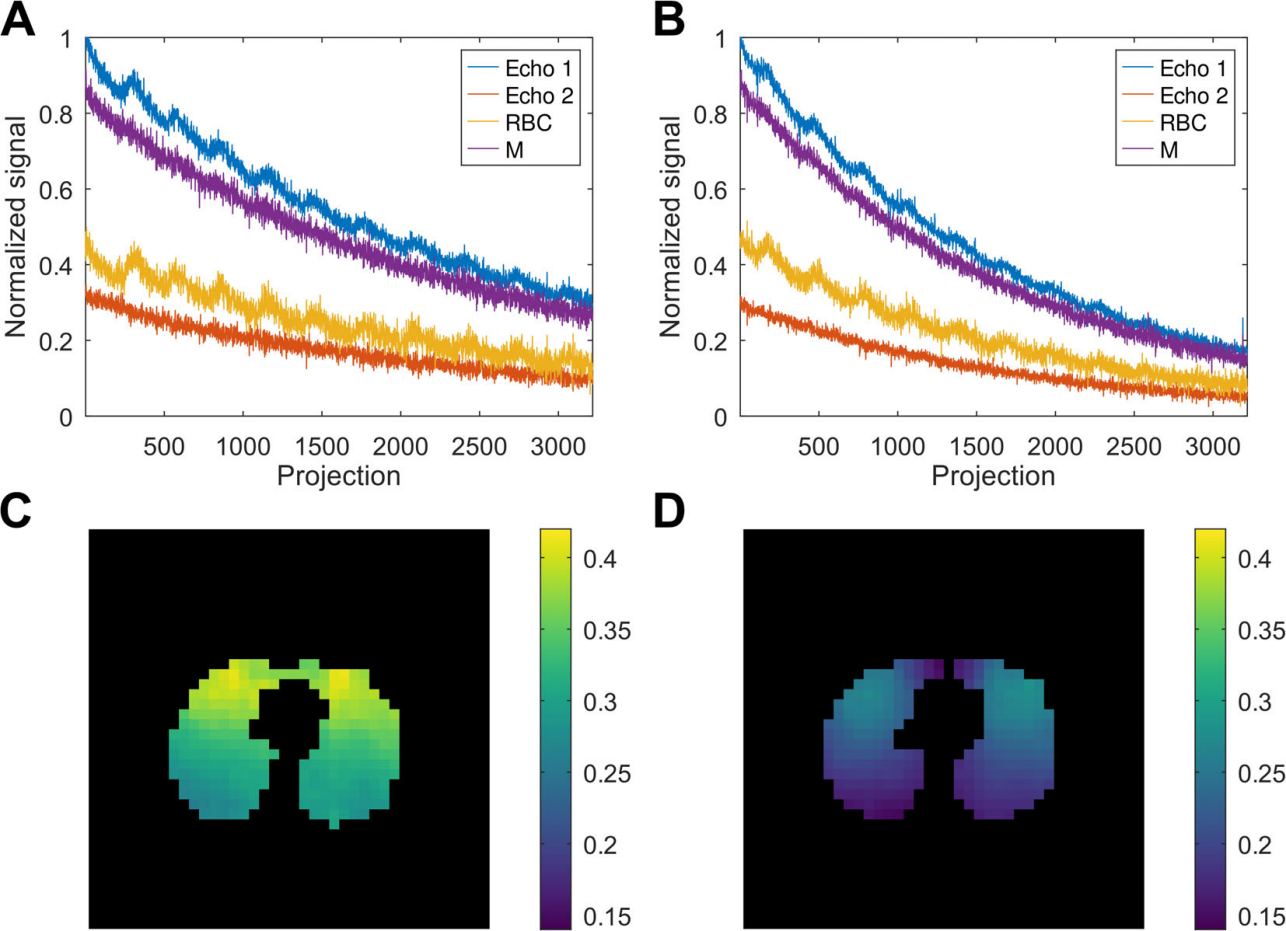
(A) Decay of signals in the k‐space center of first and second echo of bSSFP in a healthy female volunteer aged 28 years in study part A, normalized to the first data point of the first echo. Also shown are RBC and M signals separated using the constant *T*_E_ approximation. Cardiogenic oscillations are apparent mainly in the first echo and mainly in the RBC phase. (B) Analogous data for FLASH with the same high flip angle in the same volunteer. (C and D) show magnetization decay maps used for regional magnetization decay correction of the gas images in a central axial slice for bSSFP and FLASH, respectively.

Examples of decaying signals in the center of k‐space before and after chemical shift separation in bSSFP and FLASH with high flip angle are shown in Figure 6. Besides the exponential decay of the signals, cardiogenic oscillations are apparent mainly in the first echo and in the RBC phase. Also shown are decay maps used for correction of gas signal intensity. A significantly higher coefficient of variation of the local remaining signal after dissolved‐phase acquisition was found in FLASH with high flip angle compared to bSSFP, *p* = 0.001, whereas there was no significant difference between FLASH with reduced flip angle and bSSFP, *p* = 0.61, see Figure S2.

**FIGURE 7:**
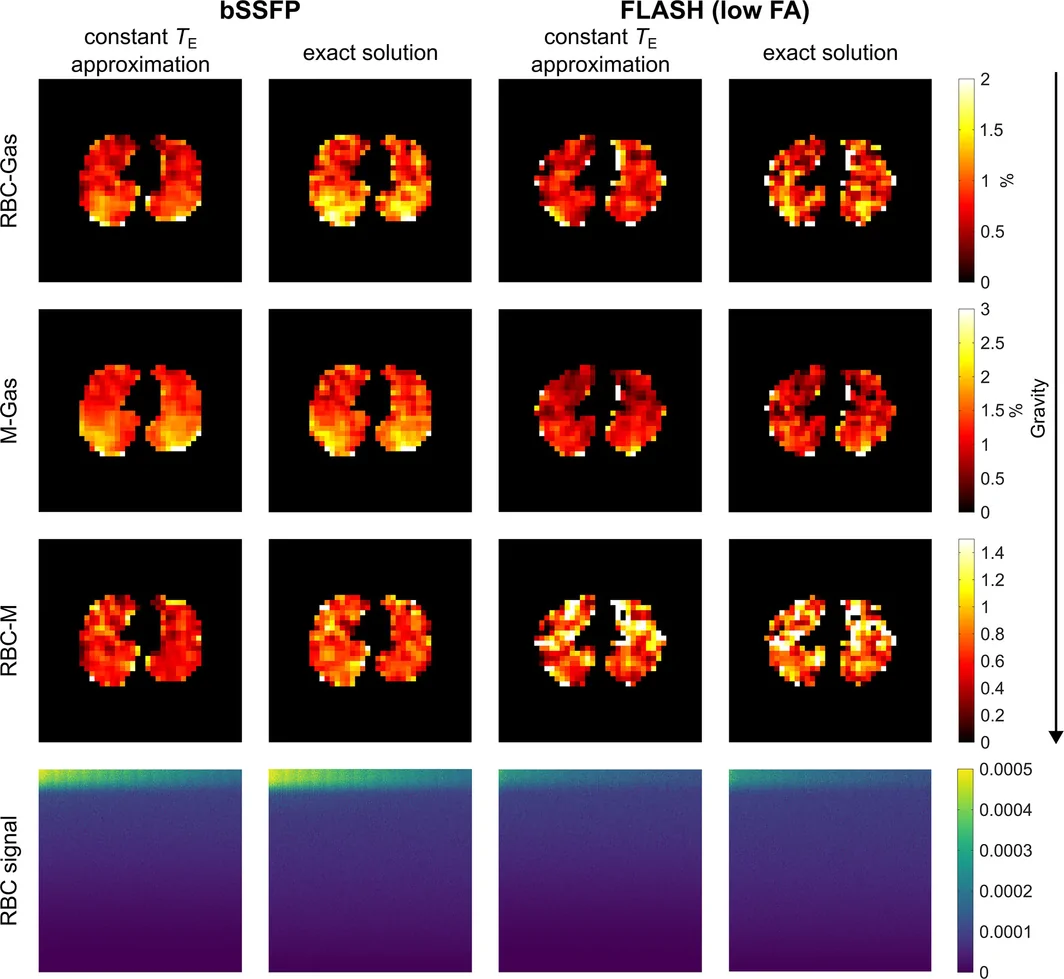
Ratio maps of ^129^Xe in RBCs and gas (first row), in M and gas (second row) and in RBC and M (third row) in axial orientation as well as RBC signal (one column per readout) after chemical‐shift separation (last row) in a 25‐year‐old male volunteer in study part B. Maps are shown for both bSSFP and FLASH with reduced flip angle and using the constant *T*_E_ approximation as well as exact solution for separation of RBC and M compartments. Somewhat higher ratios are observed for bSSFP. The constant *T*_E_ approximation is seen to introduce some bias but prevents noise amplification in outer regions of k‐space. An anteroposterior gradient following the gravitational direction (arrow) is observed in bSSFP similarly as in FLASH. Abbreviations: FA, flip angle; M, membrane; RBC, red blood cell.

Example dissolved‐phase ratio maps are shown in Figure 7 for bSSFP and FLASH with low flip angle. Maps derived using the version of Equation (2) without approximation (exact solution) are seen to suffer from noise amplification. A gravitational gradient is observed in both sequences. The absolute values are seen to differ somewhat between sequences and chemical shift separation methods. A complete volume of ratio maps in the same subject is shown in Figure S3 for bSSFP and using separation of phases with constant *T*_E_ approximation. Another example for analogous comparison of bSSFP and FLASH with high flip angle is shown in Figure S4.

**FIGURE 8:**
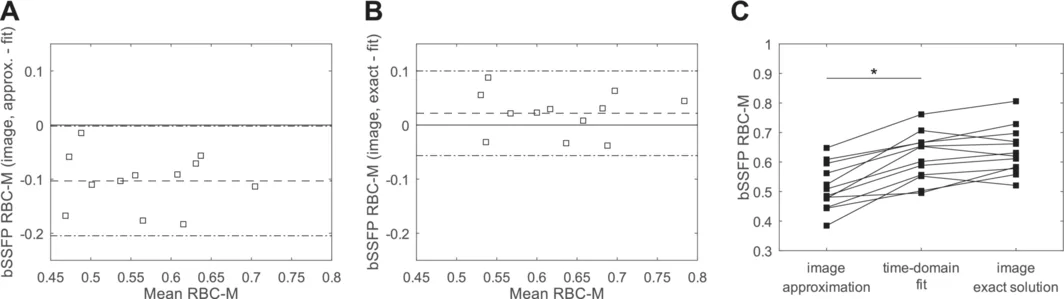
(A) Bland–Altman plot comparing RBC‐M derived from bSSFP measurements averaged over the whole lung in image space using the constant *T*_E_ approximation with results from time‐domain fits. Dashed lines indicate mean differences and dash‐dotted lines 95% limits of agreement. A bias becomes apparent. (B) The same plot is shown for the case of the exact solution for RBC‐M separation. There is no clear bias and limits of agreement are somewhat narrower. (C) Spaghetti plot for comparison of RBC‐M obtained using different methods. A significant difference is observed between RBC‐M from images using the constant *T*_E_ approximation and time‐domain fits but not between RBC‐M from time‐domain fits and images using the exact solution for compartment separation. Abbreviations: bSSFP, balanced SSFP; M, membrane; RBC, red blood cell.

Bland–Altman plots comparing bSSFP RBC‐M derived from different methods are shown in Figure 8, taking results from time‐domain fits as reference. There was good agreement of RBC‐M derived by fitting the time evolution of magnetization in the absence of spatial encoding gradients with results averaged in image space when Equation (2) was used without approximation (exact solution), with no significant difference, *p* = 0.12. The approximation of *T*_E_ as a constant during the readout is seen to introduce some bias, resulting in a significant difference, *p* < 0.001.

**FIGURE 9:**
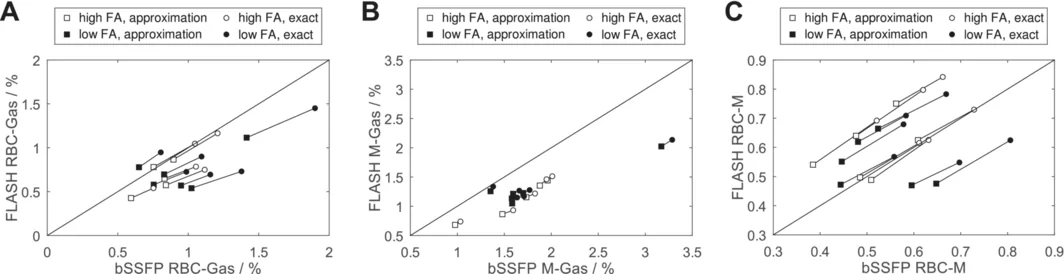
Scatter plots for descriptive comparison of imaging metrics averaged over the whole lung for (A) RBC‐Gas, (B) M‐Gas and (C) RBC‐M and different sequence types. The flip angle (FA) refers to the one used in FLASH. Data points from the same subjects are connected to guide the eye. Also shown is the line of identity. Most data points in (A) and (B) are seen to be located below the line of identity, suggesting increased values in bSSFP. No clear effect is observable in (C) despite the finding of a significant difference in results from time‐domain fits, see (Figure 5A). The constant *T*_E_ approximation is seen to affect mostly (A) RBC‐Gas and (C) RBC‐M while points from the same subject are located relatively close together in (B).

Whole lung averages of dissolved‐phase ratios from both sequences and chemical shift separation methods are compared descriptively in scatter plots in Figure 9. Again, the constant *T*_E_ approximation is seen to affect the RBC‐M ratio. Usage of bSSFP mainly seems to lead to an increase in dissolved‐gas ratios compared to FLASH.

## 4 Discussion

This study demonstrates the feasibility of hyperpolarized ^129^Xe gas‐exchange imaging with decomposition of signals into RBC and M phases using a dual‐echo bSSFP sequence. The sequence provided improved normalized SNR of the first echo compared to an analogous FLASH sequence using the same flip angle but not for a FLASH sequence with reduced flip angle, resulting in comparable depolarization. The slower apparent transverse relaxation improves SNR efficiency of the RBC‐M signal decomposition in both cases, however. While the lack of detectable SNR gain in the first echo for equivalent depolarization suggests no or only a minor SNR advantage for a potential one‐point Dixon‐type analysis, double‐ and potentially multi‐echo methods seem to benefit from bSSFP in terms of SNR efficiency. A potential benefit of using a bSSFP sequence for the established one‐point Dixon method could be reduced blurring due to slower transverse relaxation at a given readout duration.

The proposed methodology of signal decomposition using inversion of the chemical shift‐encoding matrix in Equation (2) in a dual‐echo sequence is similar to a previous multi‐echo approach [13] for ^129^Xe dissolved‐phase imaging but with the difference that the gas signal is suppressed by careful tuning of the excitation pulse such that only RBC and M signals are encoded as in [12]. Additionally, the number of echoes is reduced to two in order to reduce *T*_R_ as required for an SNR‐efficient bSSFP acquisition, which also facilitates a fully sampled acquisition at acceptable acquisition time.

The result of improved SNR provided by bSSFP imaging of the ^129^Xe dissolved phase in the lungs compared to FLASH with identical flip angle is in contrast to a previous literature report [16]. The discrepancy could potentially be due to differences in lung inflation for both sequences. We note that the lungs seemed to be at the edge of the sensitive coil region and there are notable differences in diaphragm position and lung ROI position in Figure 5 of the previous literature report, where a kidney coil was used. Another possible explanation could be the longer *T*_R_ and higher field strength used in [16], leading to off‐resonance effects.

A previous literature report found reduced SNR in dissolved‐phase imaging using a FLASH sequence with shortened *T*_R_ and preserved *T*_R,90° equiv_ and measurement time compared to a previous reference standard [10]. This may suggest the possibility to enhance SNR by using undersampling and potentially increasing *T*_R_, which may render bSSFP unfavorable. Unfortunately, the methodology of SNR quantification and the influence of iterative density compensation in the presence of varying undersampling, likely leading to variable k‐space weighting [29] seem not fully clear in [10]. We would thus argue that the SNR differences reported in [10] may at least partly be due to a degradation of the point spread function in undersampled acquisitions, besides varying SNR efficiency of the sequences. Interestingly, short and long *T*_R_ sequences with equivalent flip angle had comparable dissolved‐phase SNR for comparable undersampling in that study.

Signals in bSSFP sequences have previously been shown to form an echo at *T*_E_ = *T*_R_/2 if *T*_R_ ≪ *T*_2_ [30], which is not the case here. Our findings suggest that the time evolution of magnetization may well be described by exponentially decaying signals although the apparent time constants seem to be different compared to those from FID acquisitions. Indeed, particularly the *T*_2_* of ^129^Xe in M from fitting results seems longer compared to previous literature reports [12, 31]. Previous experiments in rat lung tissue homogenates at 9.4 T suggest values of dissolved ^129^Xe *T*_2_ in the range of 3–4 ms depending on oxygenation [7], such that the used *T*_R_ is likely roughly comparable to both *T*_2_ and *T*_2_*. Consequently, a fraction of roughly exp(−1) of initial transverse magnetization is expected to be coherently recycled in bSSFP, facilitating use of a larger flip angle at equivalent depolarization and prolonging apparent transverse relaxation, thus enhancing SNR.

Minor differences in RBC‐M between sequences could be expected since bSSFP may allow for accumulation of ^129^Xe magnetization in regions further away from the gas‐exchange area by preserving more polarization although this will likely also depend on the flip angle used. Overall, our findings indicate that dissolved‐phase ratios from the different sequences cannot be used interchangeably such that a potential future diagnostic application would likely require reference values from a standardized acquisition.

Interestingly, bSSFP images also showed noticeable signal in the region of the larynx in several subjects, which was not observed in the FLASH sequence or other previous acquisitions. This suggests the existence of an anatomic structure with long *T*_2_ of dissolved ^129^Xe whose signal is thereby enhanced in bSSFP. However, the spatial resolution seems insufficient to make a definite conclusion about the signal origin. ^129^Xe in adipose tissue has previously been found to have much longer *T*_2_ than in other observed biological compartments [32] and the larynx contains pre‐epiglottic fat. More generally, tissues with low vascularization are thought to have longer *T*_2_ of dissolved ^129^Xe due to absence of interaction with paramagnetic deoxyhemoglobin [7]. The vocal cords as a structure with high surface‐volume ratio are covered with stratified squamous epithelium [33] and thus have an avascular surface. Assignment of the signal to a particular structure or chemical shift will require further research but the phase change between the two echoes observed in Figure S1 is consistent with a single resonance approximately at the chemical shift of M. Since no intense dissolved‐phase components with long *T*_2_ exist within the lung parenchyma and the larynx is well separated, we do not expect that this has implications on the interpretation of the quantified dissolved‐phase ratios within the lung.

A limitation of the proposed bSSFP sequence is that decomposition into RBC and M signals assuming constant echo times during the readouts similar to decomposition in image space leads to biases in the estimated dissolved‐phase fractions. Similar effects can, however, be expected to arise also in multi‐echo FLASH sequences with partly asymmetric readouts [12] as well as in frequently used one‐point Dixon sequences [8]. Incorporation of the finite duration of the readout improves the accuracy of the decomposition but leads to noise amplification in outer regions of k‐space where the phase evolution of M is much less than *π*. Use of a flyback gradient and equal readout gradient polarity could be an alternative [14] but this also poses additional restrictions on readout duration, again reducing SNR efficiency and/or prolonging *T*_R_. The Bland–Altman analysis in Figure 8A suggests that the bias introduced is relatively constant, suggesting that it might be possible to correct for it. If this is confirmed in patients with lung disease, separation of phases using the constant *T*_E_ approximation may be the preferred solution.

The local gas polarization and thus gas‐phase signal acquired after dissolved‐phase acquisition during the same breathhold is influenced through xenon transfer contrast [26] which is corrected for using linear subspace reconstruction [27]. The temporal separation of dissolved and gas‐phase acquisition may still have the disadvantage of introducing a small bias in the ratio maps since the local rate of magnetization decay may be different for dissolved and gas‐phase acquisitions and thus could affect the image contrast differently, which has so far been ignored. Linear subspace reconstruction also poses restrictions on the choice of k‐space trajectory and reordering scheme which in our work thus deviates from the one proposed in [17].

The measurement time for two repetitions of the bSSFP sequence at the RBC and gas frequency is around 20 s, which may be too long for patients with lung disease. However, the acquisition of the gas image is relatively uncritical with respect to SNR at the given resolution and thus can likely be highly accelerated by means of parallel imaging or compressed sensing. Another possibility would be a separate breathhold and image registration, which may be problematic mainly due to time constraints given by polarizer throughput. It may also be possible to use interleaved acquisitions in bSSFP which, however, would require multiple phases of signal stabilization.

A well‐known limitation of bSSFP is its sensitivity to off‐resonance effects. In addition, chemical exchange may lead to asymmetries of the bands in bSSFP [34]. Future work is required to elucidate the exact off‐resonance behavior of the proposed sequence. Nonetheless, the short transverse relaxation rates of dissolved ^129^Xe within the lung suggest a relatively benign off‐resonance behavior with only small signal changes with varying off‐resonance frequency since the fraction of refocused magnetization is expected to be comparatively small.

This study has been conducted at a relatively low field strength of 1.5 T, which leads to a still acceptable required *T*_R_ of ˜2.91 ms for imaging RBC and M in neighboring pass bands. Due to the lack of commercial availability of MRI systems with lower field and multinuclear capability, future application may require the translation to higher fields like 3 T. The usefulness of double‐echo bSSFP at 3 T is an open question and shall be the matter of future research.

While we attempted to maximize SNR for a constant flip angle during dissolved‐phase acquisition, use of a variable flip angle similar to strategies previously suggested for ventilation imaging may in fact facilitate further SNR improvements [35]. This would, however, likely lead to changes of the dissolved‐phase ratios or even signals outside the parenchymal region during acquisition [36] and consequently image artifacts in the absence of correction during reconstruction.

A study limitation is the small sample size. We used discrete flip angles in an attempt to maximize SNR for bSSFP and FLASH and to isolate the contribution of magnetization decay on SNR. The investigation of a range of flip angles would be desirable for SNR optimization and there may still be small, nonsignificant differences in global and local magnetization decay between bSSFP and FLASH with reduced flip angle which could affect the SNR. Additionally, the three sequence variants were not simultaneously assessed in each subject mainly due to limitations of polarizer throughput. A further limitation is that the sequence was only tested in healthy volunteers of comparable age. Future studies should investigate the signal behavior in subjects with lung disease where reduced oxygenation is expected to lead to a change in RBC chemical shift [37, 38] which could move the RBC resonance to a bSSFP transition band. Age‐related changes previously observed in gas‐exchange imaging [39] also require studying older healthy individuals as a control group.

## 5 Conclusion

Compartment‐specific hyperpolarized ^129^Xe dissolved‐phase imaging in the lung using double‐echo bSSFP is feasible and allows for use of higher flip angles with identical depolarization compared to FLASH. Slower apparent transverse relaxation leads to improved SNR efficiency in two‐point chemical shift separation. Future work should concentrate on the application in patients with lung disease with potentially altered ^129^Xe chemical shifts.

## Funding

This work was supported by the German Center for Lung Research (DZL).

## Supporting information


**Figure S1:** Comparison of dissolved‐phase signals acquired using bSSFP and presented in Figure 2A (A) with corresponding gas signals reconstructed with an artificial off‐resonance of −3826 Hz (B) shows that the appearance in the larynx region (arrows) differs. This suggests that the signal observed in (A) is actual dissolved‐phase signal and not a consequence of contamination with gas signals from spurious excitation. The change of phase in the larynx region between the first (C) and second (D) echo relative to the receiver tuned to 3826 Hz downfield from the gas resonance is consistent with a single resonance approximately at the chemical shift of M.
**Figure S2:** Comparison of the coefficient of variation of the remaining signal within the mask encompassing the lung region in local magnetization decay correction. A significant difference is observed between FLASH with high flip angle and bSSFP, *p* = 0.001, whereas there is no significant difference in the case of FLASH with low flip angle and bSSFP, *p* = 0.61.
**Figure S3:** Complete volumes of dissolved‐phase ratio maps for the bSSFP acquisition in the 25‐year‐old male volunteer presented in Figure 7 using the constant *T*_E_ approximation.
**Figure S4:** Comparison of dissolve‐phase ratio maps obtained by bSSFP and FLASH with high flip angle analogous to Figure 7 in a 19‐year‐old female volunteer in study part A.

## Data Availability

The data that support the findings of this study are available on request from the corresponding author. The data are not publicly available due to privacy or ethical restrictions.
