# Supplementary material for: Feasibility and SNR Performance of Hyperpolarized 129Xe Gas Exchange Imaging Using a Balanced SSFP Sequence
Source: Magn Reson Med. 2026 Jul 5;96(5):2201–12. doi: 10.1002/mrm.70503 (PMC13527243; doi:10.1002/mrm.70503)
Supplement: Supplementary file 1 [file MRM-96-2201-s001.pdf]

Supporting Figures

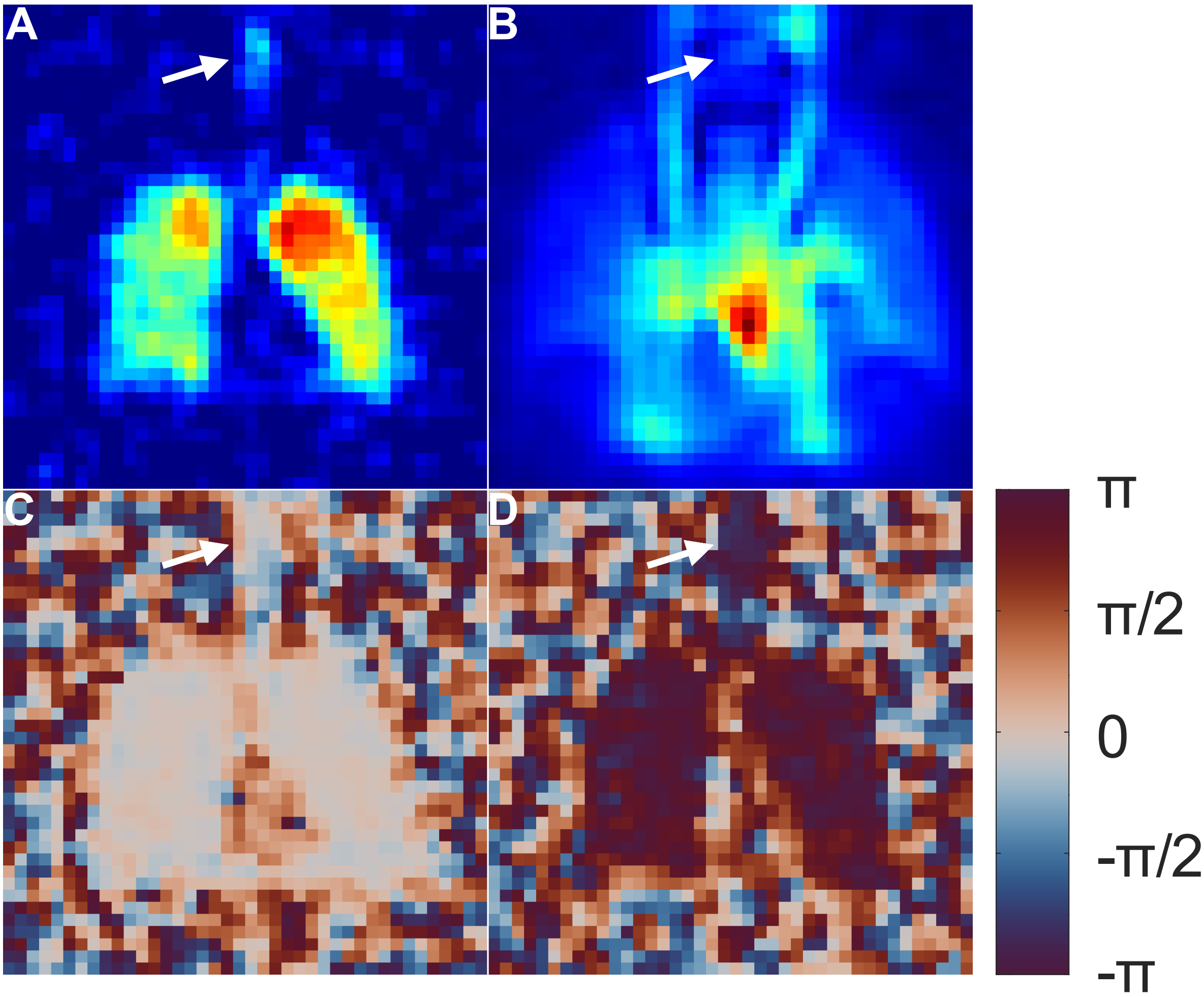

Figure S1. Comparison of dissolved-phase signals acquired using bSSFP and presented in Figure 2A (A) with corresponding gas signals reconstructed with an artificial off-resonance of −3826 Hz (B) shows that the appearance in the larynx region (arrows) differs. This suggests that the signal observed in (A) is actual dissolved-phase signal and not a consequence of contamination with gas signals from spurious excitation. The change of phase in the larynx region between the first (C) and second (D) echo relative to the receiver tuned to 3826 Hz downfield from the gas resonance is consistent with a single resonance approximately at the chemical shift of M.

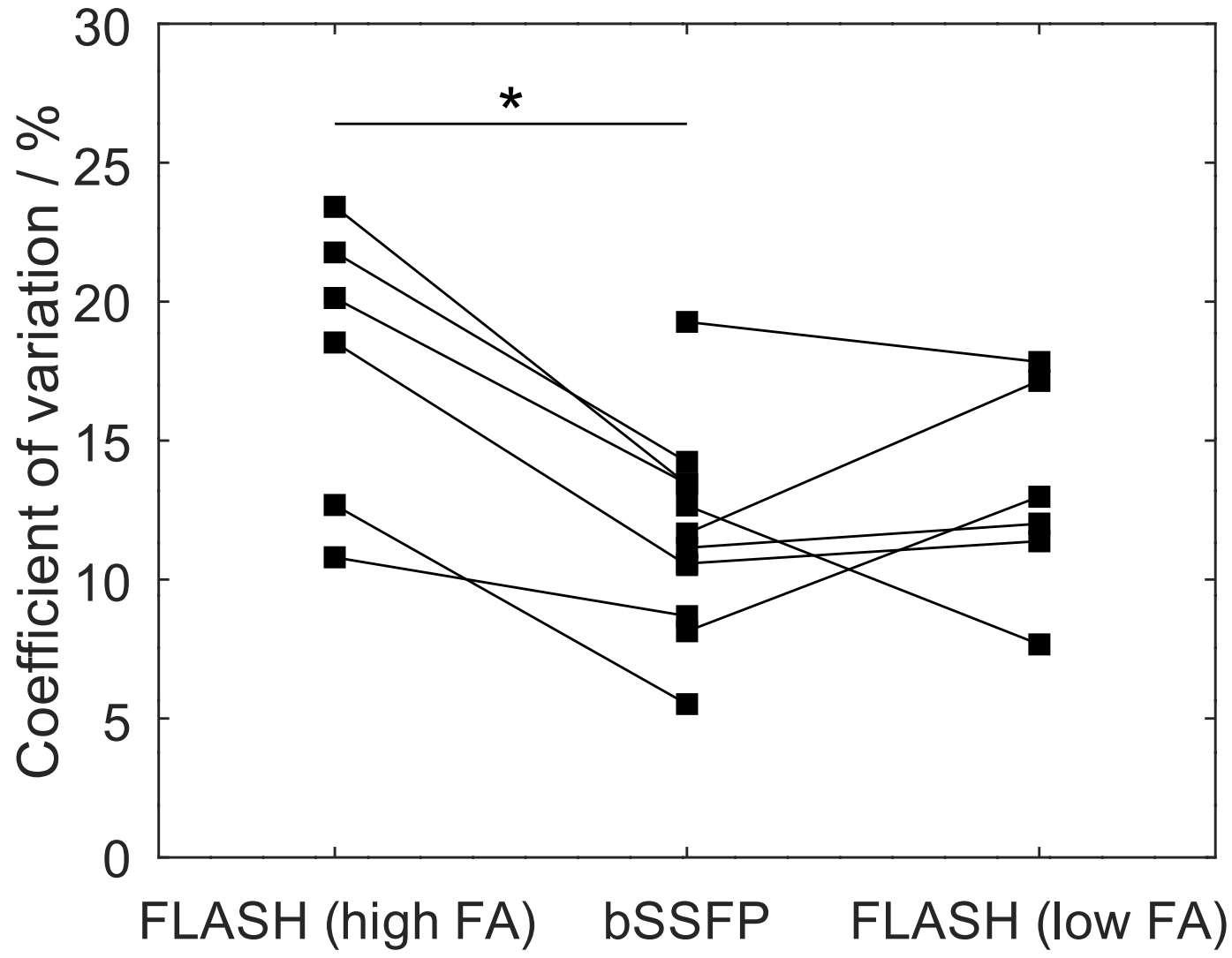

Figure S2. Comparison of the coefficient of variation of the remaining signal within the mask encompassing the lung region in local magnetization decay correction. A significant difference is observed between FLASH with high flip angle and bSSFP, $p = 0.001$, whereas there is no significant difference in the case of FLASH with low flip angle and bSSFP, $p = 0.61$.

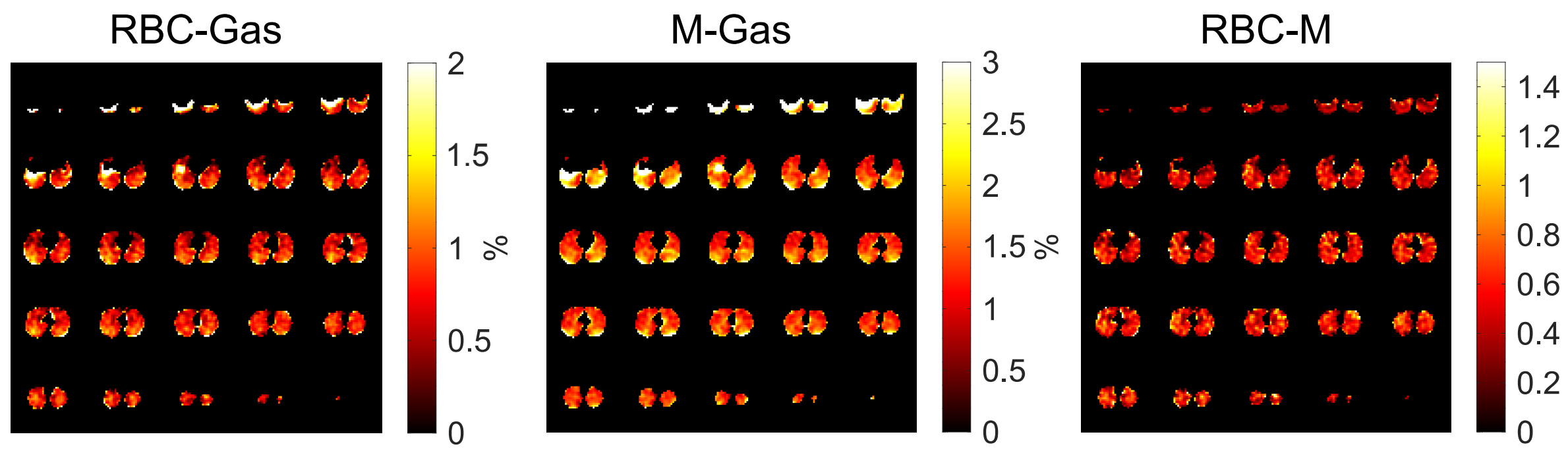

Figure S3. Complete volumes of dissolved-phase ratio maps for the bSSFP acquisition in the 25-year-old male volunteer presented in Figure 7 using the constant T_E approximation.

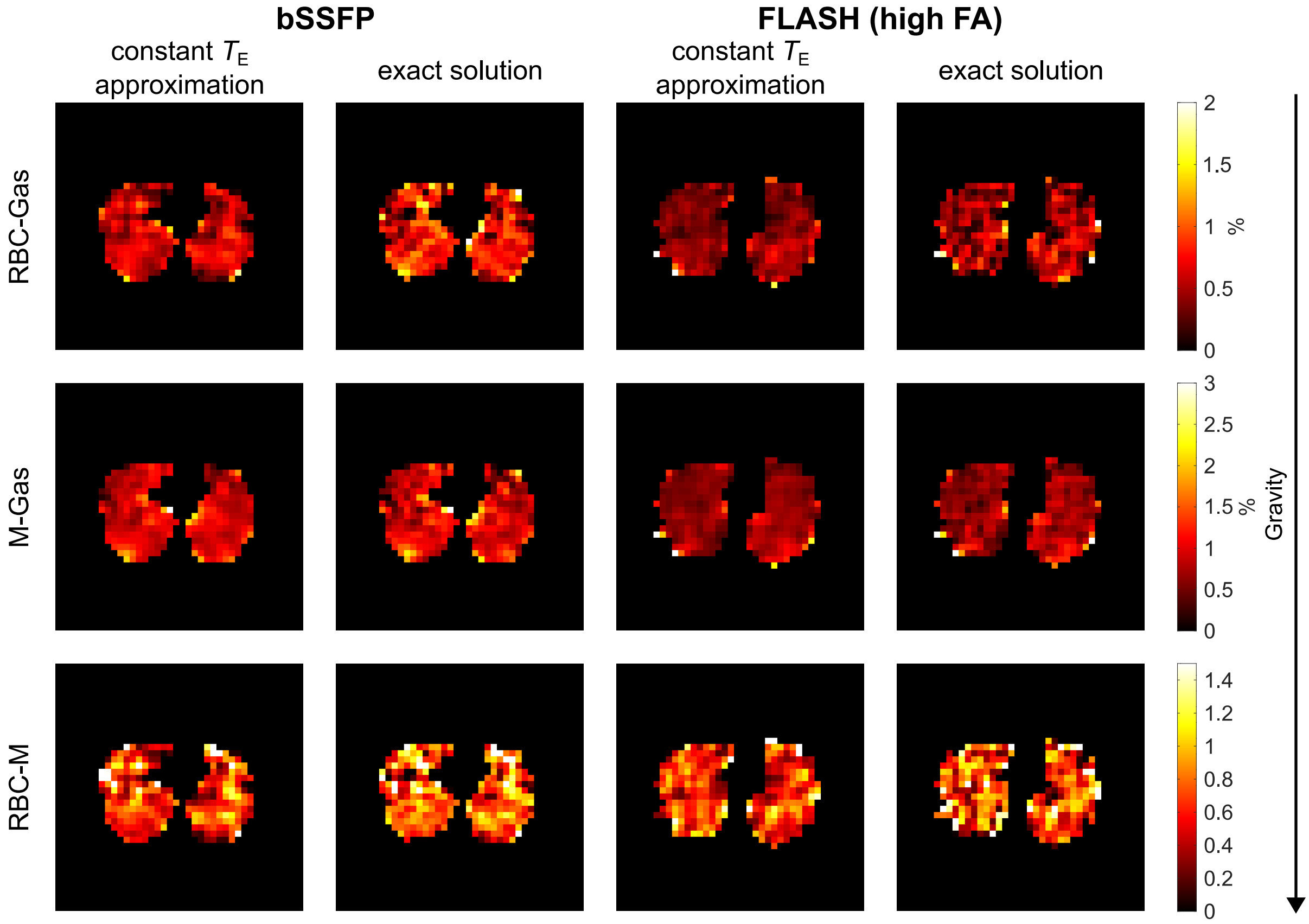

Figure S4. Comparison of dissolve-phase ratio maps obtained by bSSFP and FLASH with high flip angle analogous to Figure 7 in a 19-year-old female volunteer in study part A.
